# The influence of leader–signaled knowledge hiding on tourism employees’ work withdrawal behavior: A moderated mediating model

**DOI:** 10.3389/fpsyg.2022.1032845

**Published:** 2022-12-09

**Authors:** Anxin Xu, Haimei Zeng, Qiuqin Zheng, Xiaofeng Su

**Affiliations:** ^1^College of Economics and Management, Fujian Agriculture and Forestry University, Fuzhou, Fujian, China; ^2^Anxi College of Tea Science, Fujian Agriculture and Forestry University, Quanzhou, Fujian, China; ^3^College of Business Administration, Fujian Business University, Fuzhou, Fujian, China

**Keywords:** leader-signaled knowledge hiding, self-practiced knowledge hiding, explicit knowledge hiding, emotional exhaustion, knowledge management

## Abstract

Even though organizations encourage the dissemination of knowledge and information among organizational members, the phenomenon of knowledge hiding still exists widely in organizations. The consequences of leader-signaled knowledge hiding are more destructive to the workplace than the consequences of employees’ knowledge hiding. It is particularly necessary to explore the influence mechanism of leader-signaled knowledge hiding on employees’ work behavior. Drawing on Conservation of Resources theory, this study establishes a moderated mediation model with emotional exhaustion as a mediating variable and supervisor-subordinate *guanxi* as a moderating variable. This study focuses on the consequences of leader-signaled knowledge hiding and divides leader-signaled knowledge hiding into self-practiced knowledge hiding and explicit knowledge hiding. Based on the results of 440 questionnaires from tourism employees, it is shown that leader-signaled knowledge hiding has a positive impact on employees’ work withdrawal behavior. Specifically, leader’s self-practiced knowledge hiding has a greater direct impact on employees’ work withdrawal behavior, while leader’s explicit knowledge hiding has a greater direct impact on employees’ emotional exhaustion. Emotional exhaustion plays a key mediating role in the relationship between leader-signaled knowledge hiding (i.e., self-practiced knowledge hiding and explicit knowledge hiding) and employees’ work withdrawal behavior. Supervisor-subordinate *guanxi* significantly moderates the positive relationship between leader-signaled knowledge hiding (i.e., self-practiced hiding and explicit knowledge hiding) and employees’ emotional exhaustion. This study is an extension of previous research on knowledge hiding. The results provide a reference for leaders to deal with knowledge hiding and improve organizational knowledge management ability.

## Introduction

Even though sharing knowledge is a pervasive social norm, there is still widespread knowledge hiding in organizations (Bock et al., 2005), including the tourism industry (Arain et al., 2022). Knowledge hiding refers to individuals consciously “withhold or conceal knowledge that has been requested by another person” (Connelly et al., 2012, p.65). Knowledge hiding is a major reason for the lack of knowledge sharing in organizations. Current research, for the most part, has focused on the negative effects of employee knowledge hiding behaviors (Jha and Varkkey, 2018; Xiao and Cooke, 2019). It was found that employees’ unethical knowledge hiding has serious negative effects on organizations and individuals. For example, current researches have showed that knowledge hiding will reduce organizational performance and individual task performance (Chatterjee et al., 2021), reduce employees’ innovative work behavior (Černe et al., 2017; Jahanzeb et al., 2019), destroy inter-employee trust (Holten et al., 2016), and increase employees’ turnover intention (Serenko and Bontis, 2016). Moreover, this behavior not only has a negative impact on employees who suffer from knowledge hiding, but also the perpetrators of knowledge hiding may reduce their organizational citizenship behavior because of their sense of shame (Burmeister et al., 2019). It can be seen that knowledge hiding behavior is against ethical norms and is detrimental to organizational performance and employee development.

In practice, however, leaders may also be the perpetrators and advocate of knowledge hiding in the organization (Offergelt et al., 2019; Arain et al., 2022). This is because by explicitly signaling subordinates to hide knowledge from others, the leader can maintain the leader’s team knowledgeable and competitive (Offergelt et al., 2019). In addition, leaders themselves may also hide knowledge, which helps them maintain their authority positions and avoid being replaced by their subordinates (Butt, 2021). However, leaders’ knowledge hiding behaviors can negatively affect employee behaviors (Offergelt et al., 2019). Recent studies have found that the negative effect of leaders’ knowledge hiding behavior on employees are more severe than that of employees’ knowledge hiding behavior (Mawritz et al., 2012; Schyns et al., 2018); Offergelt et al. (2019) introduced the concept of leader-signaled knowledge hiding. His research demonstrated that when leaders expect, tolerate, or practice knowledge hiding, it has a negative effect on employees’ work attitudes and perceptions of empowerment. Arain’s series of studies confirmed that leader knowledge hiding decreases employee trust (Arain et al., 2020) and self-efficacy (Arain et al., 2019), undermines team interpersonal deviance and reduces employees’ organizational citizenship behavior (Arain et al., 2022). However, the former study has not yet distinguished the dimensions of leader-signaled knowledge hiding, and has only discussed the negative effects of it on employee’s behavior in a broad conceptual framework. The later study just extended knowledge hiding research from of the employee level to the leader level and studied the consequences of such top-down knowledge hiding for employee’s behavior.

Under the impact of the COVID-19, the tourism industry faces the challenge of providing efficient and innovative customer service, and tourism leaders must take the lead and share their own prior customer service experiences with their employees (Duro et al., 2021). Both leaders who conceal knowledge from employees and leaders who encourage people to conceal knowledge can seriously undermine the ability of travel industry employees to provide innovative customer service (Arain et al., 2022). Considering that different types of leader-signaled knowledge hiding behaviors may have different degrees of influence on employees’ attitudes and behaviors, this study synthesizes previous research and further divides leader-signaled knowledge hiding into two dimensions based on Offergelt et al. (2019) and names them as follows: self-practiced knowledge hiding (SH) and explicit knowledge hiding (EH).

The concept of self-practiced knowledge hiding (SH) is drawn on the definition of top-down knowledge hiding (Arain et al., 2020), which means the leader deliberately conceals or plays dumb to the information requested by subordinates. This behavior is classified as personal knowledge hiding behavior, and its negative effects exist between the leader and the subordinates the leader hides information from (Connelly et al., 2012; Arain et al., 2019). Explicit knowledge hiding (EH) means that leaders suggest to their subordinates that they expect and tolerate the occurrence of knowledge hiding behaviors (Offergelt et al., 2019), and its negative effects may exist within the department or the entire organization.

Personal affect and emotional state were shown to be important mediating variables in studies related to the mechanisms by which knowledge hiding behavior affects employees’ behavior (Xiao and Cooke, 2019), such as shame and guilt (Burmeister et al., 2019), self-efficacy (Arain et al., 2020), trust perception (Holten et al., 2016), and so on. In addition, relevant research in recent years have shown that emotional exhaustion is directly related to negative employee behavior in organizational stressful situations, such as absenteeism, turnover (Hobfoll et al., 2018) and reduced extra-role performance (Ain et al., 2022). According to Conservation of Resources theory, when employees suffer leader-signaled knowledge hiding behavior, they are stressed because they face a resource crisis (Hobfoll et al., 2018). This stress drives employees into a state of emotional exhaustion. Employees who are emotionally exhausted and unable to be effectively compensated may take measures such as lowering work engagement and psychologically or behaviorally withdrawing from work to avoid the threat posed by emotional exhaustion (Chong et al., 2020). Therefore, this study believes that the mediating role of emotional exhaustion in the relationship between leader-signaled knowledge hiding on employees’ work withdrawal behavior should be examined.

Most of the current research on knowledge hiding has solved the problem of knowledge hiding between employee and employee, however, research on the antecedents and mechanisms of knowledge hiding at the vertical level is still in the exploratory stage (He et al., 2021). Some scholars have started to focus on the top-down knowledge hiding of leaders in Western cultural contexts (Arain et al., 2019, 2020, 2022; Offergelt et al., 2019). However, in fact, in the context of high collectivism in China, the relationship between superiors and subordinates has a more profound impact on managing employees (Wan et al., 2021; He et al., 2022). Confucian culture focuses on interpersonal *guanxi*, and supervisor-subordinate *guanxi* is an important factor affecting the management efficiency of organizational employees (Law et al., 2000). It refers to informal and special personal interactions between supervisors and subordinates, including experience sharing, interests, and trust exchanges (Chen and Chen, 2004). Employees with a good *guanxi* with their supervisors have higher trust, commitment and emotional dependence on their supervisors (Green et al., 1996), which can alleviate the adverse effects of organizational objective factors on employees (He et al., 2019). Therefore, this study believes that when examining the relationship between leader-signaled knowledge hiding behavior and employees’ work withdrawal behavior, we should observe the moderating effect of the supervisor-subordinate *guanxi* to make a specific analysis.

Therefore, based on the Conservation of Resources theory, this study adopts a structural equation model to explore: (1) Whether leader-signaled knowledge hiding triggers employees’ work withdrawal behavior? (2) Whether emotional exhaustion plays a mediating role in the influence of leader-signaled knowledge hiding on employees’ work withdrawal behavior? (3) Can the supervisor-subordinate *guanxi* serve as a moderator between leader-signaled knowledge hiding and employees’ emotional exhaustion?

Our study contributes to the literature in two ways. First, this study divides leader-signaled knowledge hiding into two dimensions, i.e., self-practiced knowledge hiding and explicit knowledge hiding. What’s more, this study further explores the negative effects of the two types of leader-signaled knowledge hiding, respectively. This will help bring both practices to the attention of organizational behavior researchers. Second, previous research in the Chinese Confucian culture have only confirmed the effects of knowledge hiding among employees. This study expands the research level of the impact of knowledge hiding, and investigates the negative consequences of leader-signaled knowledge hiding on employees in Chinese Confucian cultural. It also reveals that in Chinese Confucian culture, supervisor-subordinate *guanxi* might mitigate the negative impact of leader-signaled knowledge hiding on employees behavior. Thus, it helps to enrich the cultural context of leader-signaled knowledge hiding study and deepen readers’ understanding of the complex processes through which leader-signaled knowledge hiding lead to employees’ work withdrawal behavior.

## Theoretical background and hypothesis development

### Conservation of resource

The Conservation of Resources (COR) proposed by Hobfoll (1989) has been widely used in organizational behavior research. COR is a stress theory, and the basic assumption is that individuals always have the motivation to protect existing resources and acquire new resources, and the actual loss and possible loss of resources will pose a threat to people. In the face of resource loss, people will go into defensive mode to protect themselves, which makes themselves defensive and aggressive (Hobfoll and Freedy, 2017). Therefore, COR theory can be used to explain people’s negative behaviors in the face of stressful events (Hobfoll et al., 2018).

According to COR theory, individuals gain or lose resources by interacting with organizational contextual factors (Hobfoll, 2002). These resources include social support, energy, and key resources (ten Brummelhuis and Bakker, 2012). In recent years, many scholars consider emotional exhaustion as the depletion of psychological resources (Halbesleben et al., 2014; Kammeyer-Mueller et al., 2016; Hobfoll et al., 2018). Depletion of psychological resources is considered to originate from objectively existing stress events in the organization (Lanaj et al., 2018), and stress events reduce the ability of employees to resist future risks. Therefore, to avoid further loss of resources, employees typically manage remaining resources strategically, tending to adopt avoidant behaviors rather than proactive behaviors (Halbesleben et al., 2014).

In this study, leader-signaled knowledge hiding is a stressful event that has an impact on employee resources. This is because when employees encounter work difficulties, the help of their supervisors and colleagues can be useful for them obtain more resources to advance the task or improve the performance of the work (Uy et al., 2017). On the contrary, the leader-signaled knowledge hiding makes employees stagnate at work, aggravates the work pressure on employees, makes them have negative emotions, and accumulates them day after day until they cause emotional exhaustion, which in turn affects their work behaviors (Ain et al., 2022). Therefore, COR is suitable for this study.

### Leader’s self–practiced knowledge hiding and work withdrawal behavior

Work withdrawal behavior refers to a series of negative reactions that employees take to avoid and resist work situations, including work distraction, lateness, absence, etc., and ultimately lead to employee resignation (Pelled and Xin, 1999). The stress events in the organization are the sources of the employees’ work withdrawal behavior (Zhu and Wu, 2020). In this study, leader-signaled knowledge hiding behavior is the source of stress.

Leader’s self-practiced knowledge hiding refers to the unethical leadership behavior of leaders who deliberately play dumb or refuse to provide knowledge resources to employees (Arain et al., 2020). In most cases, employees consider leader’s self-practiced knowledge hiding to be unethical because they inevitably need to rely on the leader’s knowledge resources to do their jobs or improve themselves (Carmeli et al., 2013). Leader’s self-practiced knowledge hiding makes employees pessimistic about their future opportunities for intellectual growth in the organization, thereby reducing employees’ organizational commitment level, that is, they are more reluctant to be part of the organization, less willing to care about colleagues, and even destroy organizational goals (Serenko and Bontis, 2016). In addition to knowledge resources, employees often expect to obtain social support resources from the organization. When employees notice the leader’s self-practiced knowledge hiding behavior, they may fear that the healthy relationships they are attempting to develop are in jeopardy, which can increase employee stress and cause withdrawal behaviors (Hobfoll and Freedy, 2017). In addition, Pereira et al. (2006) argued that individuals are more likely to engage in negative reciprocity than positive reciprocity, which indicates that employees tend to give negative feedback on the leader’s self-practiced knowledge hiding behavior and reduce their work effort. Therefore, we propose the following hypothesis:

*H1*: Leader’s self-practiced knowledge hiding is positively related to employees’ work withdrawal behavior.

### Leader’s explicit knowledge hiding and work withdrawal behavior

Leader’s explicit knowledge hiding refers to that leaders support knowledge hiding even though it goes against organizational norms. Leaders are likely to tolerate knowledge hiding or encourage employees to do so (Offergelt et al., 2019). Leader’s explicit knowledge hiding can lead to an organization’s culture of knowledge hiding (Offergelt et al., 2019). Often, while employees themselves may be the perpetrators of knowledge hiding, they perceive themselves to hide less knowledge than their colleagues around them (Serenko and Bontis, 2016). Therefore, they have a sense of crisis of resource depletion in the comparison and have confrontational psychology toward interpersonal relationships and cooperation matters in the work, and then use negative attitudes and behaviors to cope with the work, such as intensifying their knowledge hiding behaviors (Černe et al., 2014) and voluntary resignation (de Croon et al., 2004). In addition, supervisors often act as mentors and role models and have the power to reward or punish employees, which will strongly influence employees’ behavior (Mawritz et al., 2012). In an organization, even if employees are aware of the behavioral norms advocated by the company, they will still look for specific rules and signals from the behavior of their supervisors. When such rules and signals (such as explicit knowledge hiding) are inconsistent with organizational norms (such as advocating knowledge sharing), employees tend to adjust their behaviors to comply with the supervisor’s rules, increasingly hiding knowledge (Offergelt et al., 2019). However, behaviors that violate social norms harm the interests of the organization and may trigger employees’ sense of shame. To alleviate this emotional pressure, employees will increase self-directed behaviors (Burmeister et al., 2019) and show withdrawal behaviors (de Croon et al., 2004), such as avoiding communication, avoiding cooperation, denying, and avoidance. Therefore, we propose the following hypothesis:

*H2*: Leader’s explicit knowledge hiding is positively related to employees’ work withdrawal behavior.

### Leader’s self–practiced knowledge hiding and emotional exhaustion

Emotional exhaustion refers to the depletion of individual emotional resources. Emotional exhaustion is accompanied by work frustration and tension, which will lead to a decrease in individual work motivation (Maslach et al., 2001). Previous studies have shown that workplace stressful events are an important antecedent of employees’ emotional exhaustion (Kammeyer-Mueller et al., 2016). According to COR theory, leader-signaled knowledge hiding causes employees to experience resource depletion and impair their ability to acquire resources in the future (Hobfoll, 2001), thereby triggering emotional exhaustion.

This study proposes that leader’s self-practiced knowledge hiding is highly correlated with emotional exhaustion. Leaders who practice knowledge hiding may deliberately provide information that is different from what employees desire, claim not to know what employees are asking for, or promise to help them while actually doing nothing. For employees, leader’s self-practiced knowledge hiding is an act of denial and is a negative interaction. It causes mental exhaustion and low energy (Ain et al., 2022). Moreover, leader’s self-practiced knowledge hiding reduces employees’ sense of psychological empowerment, undermining their confidence in their job roles and their belief in contributing to the organization (Offergelt et al., 2019). Emotional exhaustion occurs when employees have to face high-intensity work and their remaining resources are insufficient to address them (Lanaj et al., 2018). Trougakos et al. (2015) found that fatigue makes employees more dependent on others for help in solving work problems, so they will gradually experience higher levels of mental exhaustion when help is not available and their own resources are scarce (Uy et al., 2017). Therefore, we propose the following hypothesis:

*H3*: Leader’s self-practiced knowledge hiding is positively related to employees’ emotional exhaustion.

### Leader’s explicit knowledge hiding and emotional exhaustion

In addition, this study suggests that leader’s explicit knowledge hiding is highly correlated with emotional exhaustion. In fact, employees strive to connect with others through positive social interaction, which helps them access valuable resources (Lanaj et al., 2016). However, leader’s explicit knowledge hiding implies that leaders utilize their power of position to prevent employees from sharing knowledge. When employees suffer leader’s explicit knowledge hiding behavior, they may sense they are in a lonely situation. This situation causes employees to lose knowledge resources, social support, and interpersonal resources (Hobfoll and Freedy, 2017), resulting in a sense of resource crisis. Anand and Mishra (2021) found that workplace loneliness can directly lead to emotional exhaustion. In addition, without the ability to exchange resources with colleagues through knowledge sharing, employees focus on how to protect their existing resources, ignore motivating events at work, and look for factors in the work environment that threaten their protected resources (Lanaj et al., 2018), which will consume employees’ psychological resources, leading to emotional exhaustion. Therefore, we propose the following hypothesis:

*H4*: Leader’s explicit knowledge hiding is positively related to employees’ emotional exhaustion.

### Emotional exhaustion and work withdrawal behavior

Emotional exhaustion is a state of lack of psychological resources. According to COR theory, compared with those with abundant resources, people who lack resources have a weaker ability to replenish resources and are more likely to lose resources further (Hobfoll et al., 2018), which hinders employees from better devotion to work. The pressure caused by emotional exhaustion causes employees to reduce self-control resources (Ain et al., 2022), and the value of remaining resources becomes more important (Hobfoll et al., 2018). In order to restore the remaining resources, employees will adopt withdrawal behaviors. In addition, emotionally exhausted employees have lower job satisfaction due to diminished mental energy, which in turn reduces work engagement (Pelled and Xin, 1999; Wright and Bonett, 2007). At the same time, previous empirical studies have also proved that emotional exhaustion is directly related to employee absenteeism and turnover (Van Woerkom et al., 2016; Reina et al., 2018). The study by Lanaj et al. (2018) demonstrated that emotional exhaustion causes employees who are not trusted in the organization to exhibit withdrawal behaviors. Therefore, we propose the following hypothesis:

*H5*: Employees’ emotional exhaustion is positively related to employees’ work withdrawal behavior.

### Mediating role of emotional exhaustion

Based on the viewpoints of previous section, this study proposes that emotional exhaustion plays a mediating role between leader-signaled knowledge hiding and employees’ work withdrawal behavior. Leader’s self-practice knowledge hiding and explicit knowledge hiding will directly reduce employees’ social support resources and work resources. The depletion of many resources leads to negative emotions, which in turn leads to the emotional exhaustion of employees (Offergelt et al., 2019). Emotional exhaustion causes employees to reduce their control over their self-resources, triggering a sense of crisis and ultimately self-defensive behavior (Hobfoll and Freedy, 2017), i.e., by reducing active engagement in work to protect remaining resources. Existing findings suggest that emotional exhaustion mediates the negative effects of knowledge hiding on employees’ extra-role performance (Ain et al., 2022), and that emotional exhaustion mediates the relationship between stressful work events (e.g., task frustration, abusive supervision) and employees’ withdrawal behavior (Chi and Liang, 2013; Chong et al., 2020). Therefore, we propose the following hypotheses:

*H6a*: Employees’ emotional exhaustion mediates the positive relationship between leader’s self-practiced knowledge hiding and employees’ work withdrawal behavior.

*H6b*: Employees’ emotional exhaustion mediates the positive relationship between leader’s explicit knowledge hiding and employees’ work withdrawal behavior.

### Moderating role of supervisor–subordinate *guanxi*

Although employees experience negative emotions due to stressful events in the organization, it is worth noting that not all subordinates have the same degree of emotional response to leader-signaled knowledge hiding behaviors. Unlike formal organizational relationships, supervisor-subordinate *guanxi* are informal connections between leaders and subordinates (Law et al., 2000). Leaders typically form small social circles with well-connected subordinates and exchange benefits and emotional resources with the employees in the circles (He et al., 2019). The high quality of supervisor-subordinate *guanxi* means that employees become “insiders” of the social circles. On the one hand, the “insiders” employees can have a fuller information communication and interest base with the leaders (Law et al., 2000). This will cause “insiders” employees to have assimilation psychology (Gardner et al., 2002), who will connect the process of consolidating power by leaders with the process of consolidating resources for themselves. In this situation, employees might believe that the leader’s explicit knowledge hiding behavior prevents outsiders from accessing the knowledge resources of their social circle, thus reduce resistance to knowledge hiding. On the other hand, the “insiders” employees have a stronger emotional attachment to and confidence in their leaders, and are more likely to increase positive feelings (Lau et al., 2014). Therefore, for employees who have high-quality supervisor-subordinate *guanxi*, because they perceive themselves as “insiders,” even when they perceive the leader-signaled knowledge hiding, they will rationalize the superior’s behavior from the perspective of assimilation psychology and trust, and then reduce the unethical perceptions of the superior’s behavior (Fehr et al., 2020). On the contrary, those employees with low-quality supervisor-subordinate *guanxi* are less likely to receive bonuses, promotion opportunities, and other beneficial resources from their leaders than employees who are “insiders” (Chen et al., 2009). In this context, when employees perceive the leader’s self-practiced knowledge hiding, they feel a stronger conflict of interest and develop a stronger sense of mistrust (Gan et al., 2019). When employees perceive the leader’s explicit knowledge hiding, the level of mistrust between employees increases, which creates a poor atmosphere for interpersonal interactions in the workplace (Dimotakis et al., 2011). Employees may believe themselves are more likely to suffer from organization’s knowledge hiding culture than “insider” employees (Serenko and Bontis, 2016), and thus feel cynical (Aljawarneh and Atan, 2018). Both of these situations reinforce the effect of leader-signaled knowledge hiding behavior on emotional exhaustion. Therefore, we propose the following hypothesis:

*H7a*: Supervisor-subordinate guanxi negatively moderates the positive relationship between leader’s self-practiced knowledge hiding and employees’ emotional exhaustion, that is, the positive relationship is weaker when supervisor-subordinate guanxi is higher than it is when supervisor-subordinate guanxi is lower.

*H7b*: Supervisor-subordinate guanxi negatively moderates the positive relationship between leader’s explicit knowledge hiding and employees’ emotional exhaustion, that is, the positive relationship is weaker when supervisor-subordinate guanxi is higher than it is when supervisor-subordinate guanxi is lower.

Furthermore, this study proposes a moderated mediation model to explore the influence mechanism and effect conditions of leader-signaled knowledge hiding on employees’ work withdrawal behavior. Leader-signaled knowledge hiding affects employees’ work withdrawal behavior through the mediating effect of emotional exhaustion, and this effect is mediated by the supervisor-subordinate *guanxi*. When the supervisor-subordinate’s relationship in the organization is good, the positive relationship between leader-signaled knowledge hiding and emotional exhaustion is alleviated, then the positive impact of leader-signaled knowledge hiding through emotional exhaustion on employees’ work withdrawal behavior will also be alleviated accordingly. Therefore, we propose the following hypotheses:

*H8a*: Supervisor-subordinate guanxi negatively moderates the indirect positive relationship between leader’s self-practiced knowledge hiding and employees’ work withdrawal behavior through emotional exhaustion, that is, mediated relationship is weaker when supervisor-subordinate guanxi is higher than it is when supervisor-subordinate guanxi is lower.

*H8b*: Supervisor-subordinate guanxi negatively moderates the indirect positive relationship between leader’s explicit knowledge hiding and employees’ work withdrawal behavior through emotional exhaustion, that is, t mediated relationship is weaker when supervisor-subordinate guanxi is higher than it is when supervisor-subordinate guanxi is lower.

**Figure 1:**
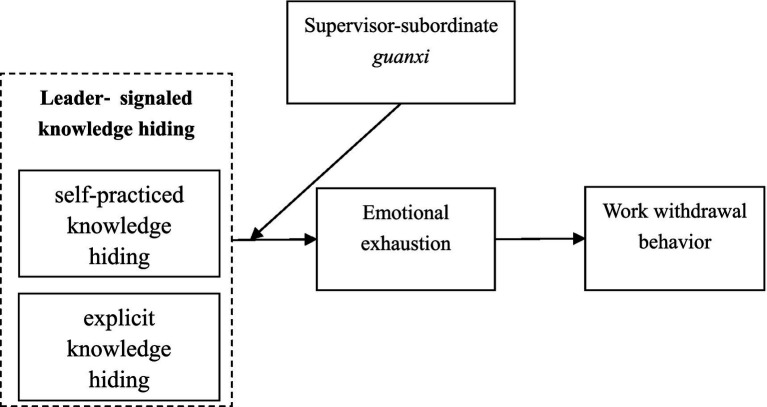
Research model.

Based on the above analysis, the theoretical model of the research is shown in Figure 1.

## Research methodology

### Design and measures

This research questionnaire is divided into two parts: the first part is the main part of the questionnaire, including the scale of each variable, and the second part is the personal information of the respondents. Measurement items of each variable in the model are from mature scales that are widely used in the relevant literature, and are appropriately modified according to expert opinions and specific employee work situations. All scales are in the form of a Likert 7-point scale.

1. Leader’s self-practiced knowledge hiding. The measure of leader’s self-practiced knowledge hiding was adopted from Connelly et al. (2012) and Offergelt et al. (2019), containing the three items “I think my supervisor sometimes hides knowledge from me,” “My supervisor never really intends to help us” and “My supervisor will say he does not understand.”
2. Leader’s explicit knowledge hiding. The measure of leader’s explicit knowledge hiding was adopted from Offergelt et al. (2019) and contained three items, “Sometimes my supervisor wants me to conceal my knowledge,” “My supervisor understands if I conceal my knowledge for some reason” and “My supervisor is tolerant when colleagues conceal their knowledge.”
3. Emotional exhaustion. Emotional exhaustion was measured using Maslach and Jackson (1981), which contains four items, “I feel emotionally drained at work,” “At the end of the day, I feel exhausted” and “When I wake up in the morning, I have to face a new day at work.”
4. Employee work withdrawal behavior. Employee work withdrawal behavior was measured using Lehman and Simpson (1992), which contains “Putting less effort into your work than you should,” “Considering leaving,” and “Spending time at work on personal matters.”
5. Supervisor-subordinate *guanxi.* The supervisor-subordinate *guanxi* was measured using Law et al. (2000), containing “I call my supervisor or visit him/her during holidays or after work,” “My supervisor would often invite me to dinner” and “I always actively share my thoughts, questions, needs and feelings with my supervisor” three items.

In the control variable section, as suggested by Bernerth and Aguinis (2016), the control variable cannot be too highly correlated with the independent variable. Zhao and Jiang (2021) proposed that potential control variables include gender, education level, tenure, etc. Therefore, in this study, demographic variables such as gender, education, age, and tenure were controlled.

The measurement scales for the key variables in this study were adopted from the English literature. To ensure the accuracy of the semantic connotation of all items in the scale and the comprehensibility of the linguistic expressions, a translation team was organized. Specifically, we invited two overseas students to join the panel to “translate and back-translate” the questionnaire items. These two students were good at both English and Chinese and their research areas were leadership and organizational behavior.

## Data collection

Our research focuses on the tourism industry for two reasons. First, the tourism industry is prone to unconventional approaches to innovation (Orfila-Sintes and Mattsson, 2009), and knowledge-oriented leadership may be a good tool to promote innovation in the tourism industry (Donate et al., 2022). Second, tourism industry is a knowledge-intensive industry (Hallin and Marnburg, 2008), it requires tourism-related practitioners to learn continuously and to share knowledge in order to provide high-quality, differentiated services. Therefore, the tourism industry is particularly vulnerable to the effects of knowledge hiding (Donate et al., 2022). We need to better understand the potential relationship between leader knowledge hiding and employee work behavior.

Considering that leader’s self-practiced knowledge hiding, leader’s explicit knowledge hiding, emotional exhaustion, and supervisor-subordinate *guanxi* are all variables of psychological perception, it is more accurate to use self-reported measures according to the recommendations of relevant research. The results of previous studies (Offergelt et al., 2019; Zhao and Jiang, 2021) have also shown that self-reported methods have high reliability and validity in measuring supervisor-subordinate’s relationship, emotional exhaustion, and knowledge hiding. Therefore, this study measures the variables involved in the model by self-reporting.

The questionnaire survey method was used in this study, and the data was collected online through a professional questionnaire platform (Credamo). The survey was conducted in July 2022. Because this study explores the influence mechanism of leader-signaled knowledge hiding on subordinates’ work behavior from the perspective of subordinates, the research object does not include leaders, but focuses on ordinary employees in the organization. To ensure that the randomly selected respondents met the requirements of this study, the following controls were performed: (1) This study sets sample filtering questions before formal questionnaire responses. Respondents working in tourism-related industries and with more than 0 years of experience were only allowed to enter the questionnaire test. (2) This study sets a confirmatory question (Gao et al., 2016), “Please select ‘1’ from the following options.” Respondents who choose other options will skip directly to the end of the questionnaire.

Before the formal survey, a small-scale preliminary survey was also conducted in this study, and a total of 50 preliminary survey questionnaires were distributed. Items with factor loading values less than 0.6 were removed using AMOS 24.0. Items with unclear and confusing meanings have been adjusted and integrated to ensure the validity of the questionnaire. A total of 450 questionnaires were completed and returned. In addition to the invalid questionnaires whose answering time was too long and too short, 440 valid questionnaires were finally obtained, and the effective recovery rate of the questionnaire was 97.77%. The demographic characteristics and basic information of the research samples are shown in Supplementary Table S1.

As shown in Supplementary Table S1, the majority of participants were female (63.9%). In terms of age distribution, the respondents were relatively young, with 53.6 and 33.6% of the respondents aged 21–30 and 31–40, respectively. This is similar to the sample distribution of previous questionnaires studying knowledge hiding (El-Kassar et al., 2022). In terms of education level, 81.6% of the respondents had a bachelor’s degree or above; the largest proportion of respondents had 5–10 years of work experience (26.4%), followed by 1–3 years (22.3%).

## Results

### Common method bias test and confirmatory factor analysis

Two methods were used to test for common method bias in this study. First, common method bias was verified using Harman’s single-factor test. Unrotated principal components analysis was performed on all question items of the study questionnaire, and the first principal component was found to explain only 37.59% of the variance, which was below the 50% criterion. Second, after adding the latent variable of common method bias using the unmeasured latent method construct (ULMC) technique, it was found that ∆CFI and ∆TFL were less than 0.1, and ∆RMSEA and ∆SRMR were less than 0.05. Therefore, common method bias would not affect the study results. In addition, this study used AMOS 24.0 for confirmatory factor analysis. As shown in Table 1, the five-factor model had the best fit indicators compared with other models, indicating that the core variables had good discriminant validity.

**Table 1:** Result of CFA and CMB of measurement models (*N* = 440).

Model	χ^2^	df	χ^2^/df	RMSEA	GFI	CLI	TFI	SRMR
SH、EH、EE、WB、GX	173.665	94	1.848	0.044	0.952	0.985	0.981	0.0303
SH + EH、EE、WB、GX	444.944	98	4.540	0.090	0.865	0.934	0.919	0.0478
SH + EH + EE、WB、GX	631.453	101	6.252	0.109	0.826	0.899	0.880	0.0592
SH + EH + EE + WB、GX	782.419	103	7.596	0.123	0.795	0.871	0.849	0.0653
SH + EH + EE + WB + GX	1311.996	104	12.615	0.163	0.692	0.770	0.735	0.1014
SH、EH、EE、WB、GX, CMV	111.663	78	1.432	0.031	0.969	0.994	0.990	0.0204

GFI, Goodness of fit index; RMSEA, Root mean square error of approximation; CFI, Comparative fit index; TLI, Tucker–Lewis Index; SH, self–practiced knowledge hiding; EH, explicit knowledge hiding; EE, emotional exhaustion; WB, work withdrawal behavior; GX, supervisor-subordinate *guanxi*.

### Reliability test

The reliability of the questionnaire was tested by CR. According to the results of the measurement model in Supplementary Table S2, the CR values were all greater than 0.7, indicating that each dimension index had sufficient reliability and internal consistency. The measurement of validity is tested by convergent validity and discriminant validity. Among them, convergent validity is mainly reflected by standardized factor loading, Z-value and AVE. The results showed that the standardized factor loadings were all greater than 0.6 and significant, and the AVEs were all greater than or close to 0.5, indicating that the scale had high convergent validity. At the same time, the correlation coefficient between any two variables is smaller than the square root of AVE of each variable itself (Supplementary Table S3), so the scale has good discriminant validity, which lays a foundation for the analysis of the structural model later.

### Measurement model testing

Using AMOS 24.0, the estimation was performed using the Maximum Likelihood method. According to Supplementary Table S4, the overall fitness test results of the model were χ^2^/df = 2.063, GFI = 0.958, AGFI = 0.935, CFI = 0.986, and RMSEA = 0.049, and all the fitness indicators of the model met the criteria, indicating that the model fits well.

### Structural equation model testing

As shown in Table 2, hypotheses *H1*–*H5* were all verified. Leader’s self-practiced knowledge hiding and explicit knowledge hiding significantly and positively affected employees’ work withdrawal behavior (*β* = 0.181, *p* < 0.05; *β* = 0.161, *p* < 0.05, respectively). When employees perceived stronger self-practiced knowledge hiding or explicit knowledge hiding of leaders, they were more likely to develop work withdrawal behavior. Thus, hypotheses *H1* and *H2* were supported. Leader’s self-practiced knowledge hiding and explicit knowledge hiding significantly and positively affected employees’ emotional exhaustion (*β* = 0.323, *p* < 0.001; *β* = 0.492, *p* < 0.001, respectively), and hypotheses *H3* and *H4* were supported. Employees’ emotional exhaustion was significantly and positively associated with employees’ work withdrawal behavior (*β* = 0.508, *p* < 0.001), and hypothesis *H5* was supported.

**Table 2:** Results of hypothesis test.

	Ustd.	S.E.	C.R.	P	Std.	Results
*H*1:SH → WB	0.122	0.054	2.261	*	0.181	Support
*H*2:EH → WB	0.131	0.067	1.953	*	0.161	Support
*H*3:SH → EE	0.323	0.045	7.183	***	0.410	Support
*H*4:EH → EE	0.492	0.055	8.967	***	0.522	Support
*H*5:EE → WB	0.437	0.083	5.288	***	0.508	Support

*p < 0.05, ***p < 0.001; Ustd., unstandardized coefficients; S.E., standard error; C.R., critical ratio; Std., standardized coefficients; SH, self–practiced knowledge hiding; EH, explicit knowledge hiding; EE, emotional exhaustion; WB, work withdrawal behavior; GX, supervisor–subordinate’s *guanxi*.

The mediating effect of this study was determined by the Bootstrapping method. Hayes (2009) suggested that Bootstrapping should be repeated at least 5,000 times for the mediation effect test. In AMOS 24.0, the sampling time was set to 5,000 times, and the confidence level was set to 95%. The results are shown in Table 3.

**Table 3:** Results of mediating effect test.

Paths	Estimate	Bias-corrected percentile method		Percentile method		Results
		Lower	Upper	Lower	Upper	
SH → EE → WB	0.141	0.079	0.227	0.074	0.220	Support
EH → EE → WB	0.215	0.121	0.335	0.118	0.327	Support
**Total effect**	0.609	0.520	0.699	0.523	0.700	
**Direct effect**	0.253	0.073	0.424	0.079	0.430	
**Indirect effect**	0.356	0.208	0.513	0.204	0.511	

Judgment was made according to the confidence interval (CI) of the indirect effect. If the CI does not contain 0, the null hypothesis is rejected, indicating that the indirect effect is not 0, and the mediating effect exists. As shown in Table 3, the indirect effect exists and is significant, indicating that the mediating effect exists; the direct effect exists and is significant, indicating that it is a partial mediating effect. The proportion of the indirect effect to the total effect was 58.46%. Thus, hypotheses H6a and H6b were supported. Moreover, the indirect effect of SH and EH are 39.61 and 60.39%, respectively.

### Moderating effect of the supervisor–subordinate *guanxi*

#### Moderating effect of supervisor–subordinate *guanxi* on leader–signaled knowledge hiding and emotional exhaustion

In order to eliminate the influence of multicollinearity, this study normalized the leader’s self-practiced knowledge hiding, leader’s explicit knowledge hiding, and emotional exhaustion, and used the standardized variables to construct interaction terms. In SPSS 24.0, the process 4.0 plug-in was used to select Model 1. Gender, age, education level, and years of work were selected as control variables, emotional exhaustion was selected as the dependent variable, and leader’s self-practiced knowledge hiding/leader’s explicit knowledge hiding was selected as the independent variable. The sampling times were set to 5,000 times, and the confidence level was set to 95%. The results are shown in Table 4.

**Table 4:** Moderating effect of supervisor–subordinate *guanxi.*

Variable type	Variables	DV: Emotional exhaustion			
		M1		M2	
		*β*	95%CI	*β*	95%CI
CV	Sex	−0.003	[−0.186, 0.179]	0.068	[−0.119, 0.255]
	Age	0.117	[−0.022, 0.256]	0.042	[−0.102, 0.185]
	Education	0.042	[−0.108, 0.193]	0.025	[−0.130, 0.179]
	Working years	−0.161***	[−0.249, −0.073]	−0.133**	[−0.224, −0.042]
IV	SH	0.595***	[0.543, 0.648]		
	EH			0.658***	[0.596, 0.720]
Moderator	GX	−0.299***	[−0.354, −0.244]	−0.188***	[−0.247, −0.129]
Interactions	SH*GX	−0.066***	[−0.096, −0.035]		
	EH*GX			−0.052**	[−0.089, −0.015]

**p < 0.01, ***p < 0.001; CV, Control variables; IV, independent variables; DV, dependent variable.

According to Table 4, the regression coefficients of interaction terms on emotional exhaustion are all significant (*β* = −0.066, *p*<0.001; *β* = −0.052, *p* < 0.01). Thus, *H7a* and *H7b* are supported.

**Figure 2:**
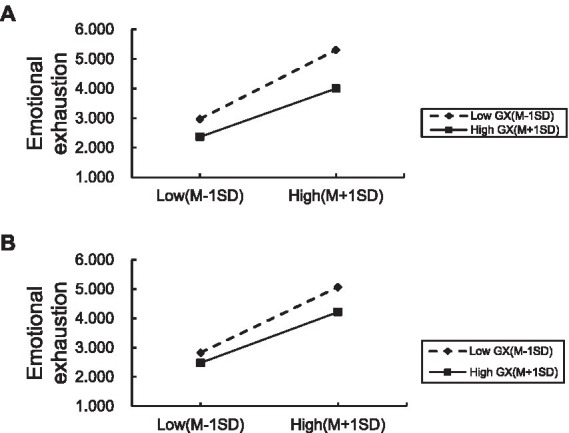
**(A,B)** Moderating effect of supervisor-subordinate *guanxi*.

As suggested by Aiken et al. (1991), this study plotted the interaction effects as one standard deviation above and below the mean, respectively. As shown in Figures 2A,B compared to the low supervisor-subordinate’s *guanxi*, the high supervisor-subordinate’s *guanxi* can alleviate the positive effects of leader’s self-practiced knowledge hiding and leader’s explicit knowledge hiding on employees’ emotional exhaustion. Therefore, hypotheses *H7a* and *H7b* were further supported.

### Moderated mediating effect

Model 7 in the Process 4.0 plug-in was used to test the moderated mediating effect, and the results are shown in Table 5.

**Table 5:** Results of moderated mediating effect test.

Moderator	Path1: SH → EE → WB				
	Levels	*β*	S.E.	LLCI	ULCI
GX	Low GX(M-1SD)	0.298	0.034	0.235	0.367
	Middle GX (M)	0.254	0.028	0.200	0.310
	High GX(M + 1SD)	0.209	0.026	0.161	0.260
	Differences	−0.045	0.010	−0.067	−0.026
	**Path2: EH → EE → WB**				
	Low GX(M-1SD)	0.297	0.039	0.220	0.373
	Middle GX (M)	0.264	0.034	0.198	0.331
	High GX(M + 1SD)	0.231	0.034	0.168	0.299
	Differences	−0.033	0.014	−0.062	−0.008

According to Table 5, in the path of leader’s self-practiced knowledge hiding → emotional exhaustion → employees’ work withdrawal behavior, when the supervisor-subordinate *guanxi* was low, the indirect effect value was 0.298, and the 95% CI was [0.235, 0.367]; when the supervisor-subordinate *guanxi* was the mean value, the indirect effect value was 0.254, and the 95% CI was [0.200, 0.310]; when the supervisor-subordinate *guanxi* was high, the indirect effect value was 0.209, and the 95% CI was [0.161, 0.260]. At different degrees of supervisor-subordinate *guanxi*, the 95% CI of the difference in indirect effects was [−0.067, −0.026], and the indirect effect difference reached a significant level. This indicates that the supervisor-subordinate *guanxi* significantly moderates the mediating role of emotional exhaustion in the relationship between leader’s self-practiced knowledge hiding and employees’ work withdrawal behavior. Therefore, hypothesis H8a is supported. Similarly, in the path of leader’s explicit knowledge hiding → emotional exhaustion → employees’ work withdrawal behavior, the difference in the 95% CI of the indirect effect was [−0.062, −0.008], reaching a significant level. This indicates that the supervisor-subordinate *guanxi* significantly moderates the mediating role of emotional exhaustion in the relationship between leader explicit knowledge hiding and employees’ work withdrawal behavior. Therefore, hypothesis *H*8b is supported.

## Conclusion and discussion

### Discussion

Knowledge is the key to an organization’s competitive advantage, and its management plays a vital role in promoting organizational success and maintaining long-term sustainable development (Pereira et al., 2018). In the past few years, many enterprises have adopted various kinds of knowledge management systems to encourage the dissemination of knowledge and information among the members of the organization, but the phenomenon of knowledge hiding still exists widely in the organization (Connelly et al., 2012). And the consequences of leader-signaled knowledge hiding can be more damaging to the workplace than employees’ knowledge hiding (Offergelt et al., 2019; Arain et al., 2020, 2021, 2022). Considering the damage of leader-signaled knowledge hiding behavior to organizational performance and employees, it is particularly necessary to explore the influence mechanism of leader-signaled knowledge hiding and employees’ work behavior. Based on the theory of Conservation of Resources, this study proposes a mediating effect model of leader-signaled knowledge hiding → emotional exhaustion → employees’ work withdrawal behavior, and a moderating effect model in which the supervisor-subordinate *guanxi* is the moderating variable. The following conclusions can be obtained from the results:

First, the leader-signaled knowledge hiding positively influences employees’ work withdrawal behavior. This result is consistent with the conclusions from Jiang et al. (2019), Singh (2019) and Offergelt et al. (2019). Knowledge hiding reduces employees’ job performance, increases turnover, and produces counterproductive behavior. Knowledge hiding is an uncooperative, pro-social, and unethical behavior that goes against the knowledge-sharing advocated in an organization (Serenko and Bontis, 2016; Bavik et al., 2017; Men et al., 2020). Under the interactive influence of leader-signaled knowledge hiding and knowledge sharing norms, employees may experience conflicts between leadership authority and organizational norms, which can easily lead to stress, tension, and negative work attitudes (de Croon et al., 2004; Zhang and Min, 2021). Offergelt et al. (2019) considered leader-signaled knowledge hiding as a destructive leader behavior. Previous studies have shown that unethical leadership or supervisory behavior, i.e., abusive supervisory (Mackey et al., 2017) and self-serving leadership (Peng and Wei, 2018, 2020), has a negative impact on followers or work attitudes and behaviors. Therefore, leader-signaled knowledge hiding will penetrate down through the organization and ultimately affect the work behavior of subordinates (Jahanzeb et al., 2019; Mahmood et al., 2021).

Second, leader’s self-practiced knowledge hiding has a greater direct impact on employees’ work withdrawal behavior, while the leader’s explicit knowledge hiding has a greater indirect impact on employees’ work withdrawal behavior through emotional exhaustion. Most of the existing research focuses on the knowledge hiding between employees, and only a few studies have explored the knowledge hiding relationship between leaders and employees (Offergelt et al., 2019; Arain et al., 2020, 2022). Based on the research by Offergelt et al. (2019), this study made a distinction between leader-signaled knowledge hiding, i.e., leader’s self-practiced knowledge hiding and leader’s explicit knowledge hiding. Compared with leader’s self-practiced knowledge hiding, leader’s explicit knowledge hiding could be a more unethical, which is more likely to cause emotional exhaustion (Qin et al., 2021). Leader’s explicit knowledge hiding signals that hidden knowledge is expected and tolerated in the organization, and actually conveys to their subordinates a concept of working in isolation. This type of poor interpersonal interactions in the workplace can easily trigger negative emotional states in employees, and such negative emotional experiences increase the likelihood of emotional exhaustion (Dimotakis et al., 2011).

Third, emotional exhaustion played a key mediating role in the relationship between leader-signaled knowledge hiding and employees’ work withdrawal behavior, with the mediating effect accounting for 39.61 and 60.39%, respectively. According to the stressor-emotion model (Spector and Fox, 2005; Fox and Spector, 2006), in the relationship between stressors and employees’ work withdrawal behavior, emotions play a role in linking the past and the future, that is, when employees feel stressed, they generate emotions, which in turn stimulate behaviors. Emotional exhaustion is described as the sense of exhaustion and exhaustion of emotional and physical resources that employees feel at work due to their devotion to a lot of emotional resources (Maslach et al., 2001). Previous studies have confirmed that leader-signaled knowledge hiding will give employees a sense of pressure, tension and frustration. When employees cannot replenish emotional resources in time, employees will experience emotional exhaustion (De Croon et al., 2004; Leat and El-Kot, 2009; Zhao and Jiang, 2021). According to the Conservation of Resources theory, when employees feel that their emotional resources are exhausted, they will show a series of negative work attitudes and behaviors, such as reducing work effort, perfunctory, and even adopting escape strategies to protect and maintain their emotional resources. In the long run, they will be tempted to leave.

Fourth, the supervisor-subordinate *guanxi* has a negative moderating effect on the relationship between leader-signaled knowledge hiding and employees’ emotional exhaustion. Specifically, a high significantly moderates the positive effect of leader-signaled knowledge hiding on employees’ emotional exhaustion, and employees with high-level supervisor-subordinate *guanxi* are less likely to experience emotional exhaustion. Conversely, low-level supervisor-subordinate *guanxi* do not have a moderating effect. In *guanxi*-oriented Chinese society, people generally value informal social exchange relations between private individuals (Su et al., 2007). The results of this study confirmed that the effect of leader-signaled knowledge hiding on employees’ emotional exhaustion was influenced by the unique situational factor of supervisor-subordinate *guanxi.* Unlike formal organizational relationships, supervisor-subordinate *guanxi* are informal connections between leaders and subordinates (Law et al., 2000). High-quality supervisor-subordinate *guanxi* can lead to higher levels of information exchange, trust, competence, commitment, role clarity, higher job satisfaction, and lower job stress (Abdullah et al., 2019; Lee and Zhong, 2020). A good communication and exchange *guanxi* with the supervisor can make it easier for the subordinate to be accepted by the supervisor as an “insider,” thereby reducing the pressure and tension caused by the leader-signaled knowledge hiding (Fida et al., 2015). That is, high-quality supervisor-subordinate’s *guanxi* can increase subordinates’ tolerance level for leader-signaled knowledge hiding, thereby greatly reducing their emotional exhaustion. Further, the supervisor-subordinate’s *guanxi* negatively moderated the direct and positive mediating effect of employees’ emotional exhaustion on leader-signaled knowledge hiding and employees’ work withdrawal behavior. When the supervisor-subordinate’s *guanxi* in the organization is good, the positive *guanxi* between leader-signaled knowledge hiding and emotional exhaustion is alleviated, then the positive impact of leader-signaled knowledge hiding through emotional exhaustion on employees’ work withdrawal behavior will also be alleviated accordingly.

### Theoretical implications

First, this study breaks through the limitation of previous studies that mainly focus on employees’ knowledge hiding. First of all, there have been a lot of studies on the cause factors of knowledge hiding behavior, but there are still few studies on its effect factors (Burmeister et al., 2019; El-Kassar et al., 2022). Previous studies have mainly focused on the antecedent variables of knowledge hiding, and explored the conditions that cause knowledge hiding, such as knowledge characteristics (Connelly et al., 2012; Hernaus et al., 2018), individual level (Peng, 2013; Pan et al., 2018; Singh, 2019), team level (Černe et al., 2014; Men et al., 2020; Zhao and Jiang, 2021), and organizational context (Aljawarneh and Atan, 2018; Abubakar et al., 2019). This study focuses on the outcome variables of knowledge hiding. Second, while there are few empirical studies (Černe et al., 2014, 2017) examining the consequences of knowledge hiding at the horizontal level (between employees and employees), the consequences at the vertical level (between supervisors and employees) have yet to be explored (Connelly and Zweig, 2015; Arain et al., 2022). By focusing on leader-signaled knowledge hiding, this study helps to expand the literature on knowledge hiding and unethical leadership/supervisory behavior.

Second, this study broadens the research context of knowledge hiding. Xiao and Cooke (2019) called for more cross-cultural comparative studies in a review exploring the extent to which knowledge hiding is harmful to organizations. Compared with developed countries, emerging markets have received less attention from researchers (Lu et al., 2010). This study takes China as the research context and introduces supervisor-subordinate’s *guanxi* as a moderating variable. Chinese society is known for its high collectivism, high traditionality, and large power distance. Many scholars believe that “*guanxi*” has a special significance in managing Chinese employees (e.g., Law et al., 2000; Wan et al., 2021). The overlap between work and social relations is much more prevalent in China than in other countries (Su et al., 2022). The superior-subordinate *guanxi* in the Chinese context is usually established through non-work factors. It is an integration of contractual and status relationships with distinct hierarchical differences. Therefore, it is of great theoretical value and practical guiding significance to explore the influence mechanism of leader-signaled knowledge hiding on employees’ work withdrawal behavior in the context of Chinese culture.

Third, this study expands scholars’ research on employees’ work withdrawal behavior. For the study of employees’ work withdrawal behavior, scholars mainly analyzed the influence of three types of factors on work withdrawal behavior, including the Big Five individual characteristics (LeBreton et al., 2004), the abusive management (Sulea et al., 2013), and the organizational justice (Cole et al., 2010). All three types of factors trigger employees’ work withdrawal behaviors through people’s emotional processes. However, existing research has not addressed the relationship between leader-signaled knowledge hiding and employees’ work withdrawal behavior. In fact, previous studies briefly expounded that knowledge hiding may lead to emotional exhaustion. Based on this, this study classifies leader-signaled knowledge hiding as leader’s self-practiced knowledge hiding and explicit knowledge hiding, and explores the influence mechanism of different types of leader-signaled knowledge hiding on the employees’ work withdrawal behavior. This broadens the research perspective of employees’ work withdrawal behavior to a certain extent and enriches the existing results.

### Practical implications

Understanding when and how leader-signaled knowledge hiding affects employee work behavior has practical implications. This research can provide a reference for leaders to deal with knowledge hiding and improve organizational knowledge management capabilities. Most of these implications are general and can inspire all industries.

First and foremost, leaders need to pay attention to the important role they play in organizational knowledge management. On the one hand, they need to be clear that their knowledge-hiding behavior affects the motivation of their subordinates. Therefore, leaders should take the initiative to share knowledge and help their subordinates develop relevant skills. When facing employees’ knowledge requests, they should give them timely and clear responses. Organizations can set up corresponding rules and regulations to manage rewards and punishments for leaders’ knowledge behaviors and set clear criteria for acceptable and unacceptable knowledge behaviors (Arain et al., 2022). On the other hand, leader’s explicit knowledge hiding behaviors can also frustrate subordinates’ work motivation. Leaders should create a corporate atmosphere of knowledge sharing by opening communication channels and organizing open group discussions. Leaders should also encourage subordinates to express their ideas, positions, and feelings (Arain et al., 2020). Organizations need to enhance organizational ethics training for leaders to ensure that team leaders adopt healthy leadership behaviors toward their subordinates (Arain et al., 2022).

Second, emotional exhaustion is an intermediate mechanism for leader-signaled knowledge hiding to lead to employees’ work withdrawal behavior. Therefore, closing the channel of excessive emotional resource consumption is one of the ways to inhibit employees’ work withdrawal behavior. From the entreprise level, enterprise managers can consider redesigning and assigning work, increasing empowerment, and formulating appropriate compensation policies to improve employees’ self-esteem, sense of belonging, and positive behavioral intentions, and ultimately improve employees’ personal resources (Cole et al., 2010). Since tourism work is characterized by long, unsocial hours, including night and weekend shifts (Chan et al., 2019), enterprise managers can also set up special rest areas and supply areas, implement flexible working systems, and improve vacation systems to help eliminate employee dissatisfaction (Grobelna, 2021). From the leader level, leaders should guide subordinates to have a good evaluation of themselves and recognize their own emotions and importance to the department and the entire organization, so as to promote subordinates to have a more positive work attitude. From the employee level, employees need to view the dilemmas they face positively and adopt constructive responses rather than responding negatively to the leaders’ knowledge hiding behavior by blindly following them (Zhao and Jiang, 2021).

Finally, high-quality supervisor-subordinate’s *guanxi* can alleviate the negative effects of leader-signaled knowledge hiding. For the organization, it should focus on both leaders and subordinates (Zhao and Jiang, 2021). Strengthen the skills training of supervisor leadership and subordinate interpersonal relationship, and solidly build a harmonious structure of supportive and trusting supervisor-subordinate *guanxi*. Various activities can also be held to increase the opportunities for communication between leaders and subordinates to enhance interpersonal interaction (Su et al., 2022). For HR, create conditions to moderate the negative impact of destructive leadership behaviors and weaken the impact of knowledge hiding behaviors through personnel selection of leaders with better moral quality and good communication skills. For employees, it is important to pay attention to the positive role of superior-subordinate *guanxi*, master the skills of handling interpersonal relationships, and take the initiative to enhance communication with superiors.

### Limitations and future prospects

First, this study uses employee self-assessment to measure the main variables. Although it has passed the common method bias test, there may still be a certain degree of self-attribution bias. Therefore, follow-up research should try to collect multi-source bias or paired sample data. Secondly, this study only partially reveals the process “black box” of leader-signaled knowledge hiding on employees’ work behavior. Although the emotional exhaustion introduced in this study has been proved to be a powerful mediating variable, the literature on organizational behavior points out that emotion and cognition are the two core elements that jointly drive employee behavior (Lee and Allen, 2002). Therefore, in the future, the emotional process mechanism and the cognitive process mechanism can be placed in the same theoretical model to explore the influence of leader-signaled knowledge hiding.

## Data availability statement

The original contributions presented in the study are included in the article/Supplementary material, further inquiries can be directed to the corresponding author.

## Ethics statement

The informed consent of the participants was implied through survey completion. An ethics approval was not required as per applicable institutional and national guidelines and regulations.

## Author contributions

HZ and QZ: data curation. AX, XS, and QZ: methodology, and writing-review and editing. AX, HZ, XS, and QZ: writing-original draft. All authors contributed to the article and approved the submitted version.

## Funding

This research was supported by the National Social Science Foundation of China (No.21BGL148): “Spatial – Temporal Differentiation and Management Response of China’s Tourism Industrial Ecologization.”

## Conflict of interest

The authors declare that the research was conducted in the absence of any commercial or financial relationships that could be construed as a potential conflict of interest.

## Publisher’s note

All claims expressed in this article are solely those of the authors and do not necessarily represent those of their affiliated organizations, or those of the publisher, the editors and the reviewers. Any product that may be evaluated in this article, or claim that may be made by its manufacturer, is not guaranteed or endorsed by the publisher.

## Supplementary material

The Supplementary material for this article can be found online at: https://www.frontiersin.org/articles/10.3389/fpsyg.2022.1032845/full#supplementary-material
