# Supplementary material for: The influence of leader–signaled knowledge hiding on tourism employees’ work withdrawal behavior: A moderated mediating model
Source: Front Psychol. 2022 Dec 9;13:1032845. doi: 10.3389/fpsyg.2022.1032845 (PMC9784470; doi:10.3389/fpsyg.2022.1032845)
Supplement: Supplementary file 1 [file Data_Sheet_1.docx]

Supplementary Material

**TABLE S1** Descriptive statistics characteristics of the sample (N = 440).

| **Variables** | **Definition** | **Frequency** | **Percentage (%)** |
| --- | --- | --- | --- |
| Sex | Male | 159 | 36.1 |
|  | Female | 281 | 63.9 |
| Age | 21 - 30 | 236 | 53.6 |
|  | 31 - 40 | 148 | 33.6 |
|  | 41 - 50 | 29 | 6.6 |
|  | 51 - 60 | 25 | 5.7 |
|  | > 60 | 2 | 0.5 |
| Education | Junior college and below | 81 | 18.4 |
|  | Undergraduate | 311 | 70.7 |
|  | Master | 43 | 9.8 |
|  | PhD | 5 | 1.1 |
| Working years | (0, 1) | 70 | 15.9 |
|  | [1, 3) | 98 | 22.3 |
|  | [3, 5) | 62 | 14.1 |
|  | [5, 10) | 116 | 26.4 |
|  | ≥ 10 | 94 | 21.4 |

**TABLE S2** Results of measurement model analysis.

| **Variables** | **Items** | **Ustd.** | **S.E.** | **Z-value** | **P** | **Std.** | **SMC** | **CR** | **AVE** |
| --- | --- | --- | --- | --- | --- | --- | --- | --- | --- |
| SH | SH1 | 1.000 |  |  |  | 0.850 | 0.723 | 0.867 | 0.686 |
|  | SH2 | 0.893 | 0.051 | 17.654 | *** | 0.780 | 0.608 |  |  |
|  | SH3 | 1.033 | 0.055 | 18.794 | *** | 0.853 | 0.728 |  |  |
| EH | EH1 | 1.000 |  |  |  | 0.811 | 0.658 | 0.907 | 0.765 |
|  | EH2 | 1.136 | 0.052 | 21.900 | *** | 0.899 | 0.808 |  |  |
|  | EH3 | 1.138 | 0.052 | 22.067 | *** | 0.910 | 0.828 |  |  |
| EE | EE1 | 1.000 |  |  |  | 0.819 | 0.671 | 0.918 | 0.738 |
|  | EE2 | 1.285 | 0.057 | 22.617 | *** | 0.893 | 0.797 |  |  |
|  | EE3 | 1.320 | 0.058 | 22.928 | *** | 0.902 | 0.814 |  |  |
|  | EE4 | 1.161 | 0.058 | 19.958 |  | 0.819 | 0.671 |  |  |
| WB | WB1 | 1.000 |  |  |  | 0.738 | 0.545 | 0.839 | 0.636 |
|  | WB2 | 1.053 | 0.070 | 15.003 | *** | 0.786 | 0.618 |  |  |
|  | WB3 | 1.154 | 0.076 | 15.184 | *** | 0.864 | 0.746 |  |  |
| GX | GX1 | 1.000 |  |  |  | 0.821 | 0.674 | 0.873 | 0.696 |
|  | GX2 | 0.938 | 0.052 | 18.072 | *** | 0.804 | 0.646 |  |  |
|  | GX3 | 1.096 | 0.058 | 18.980 | *** | 0.876 | 0.767 |  |  |

*** *p* < 0.001; CR, composite reliability; AVE, average variance extracted; SH, self‐practiced knowledge hiding; EH, explicit knowledge hiding; EE, emotional exhaustion; WB, work withdrawal behavior; GX, supervisor-subordinate *guanxi*.

**TABLE S3** Results of discriminant validity test.

| **Variables** | **Mean** | **SD** | **AVE** | **GX** | **WB** | **EE** | **EH** | **SH** |
| --- | --- | --- | --- | --- | --- | --- | --- | --- |
| Sex | 1.640 | 0.481 | **-** |  |  |  |  |  |
| Age | 1.660 | 0.869 | **-** |  |  |  |  |  |
| Education | 1.940 | 0.569 | **-** |  |  |  |  |  |
| Working years | 3.150 | 1.400 | **-** |  |  |  |  |  |
| GX | 4.286 | 1.598 | 0.696 | **0.834** |  |  |  |  |
| WB | 3.301 | 1.222 | 0.636 | -0.368 | **0.797** |  |  |  |
| EE | 3.692 | 1.521 | 0.738 | -0.524 | 0.789 | **0.859** |  |  |
| EH | 3.770 | 1.511 | 0.765 | -0.414 | 0.721 | 0.833 | **0.875** |  |
| SH | 4.184 | 1.667 | 0.686 | -0.216 | 0.712 | 0.806 | 0.757 | **0.828** |

The items on the diagonal represent the square roots of the AVE; off-diagonal elements are the correlation estimates.

**TABLE S4** Fitting results of model.

| **Index** | **Criteria** | **Model fit** | **Result** |
| --- | --- | --- | --- |
| χ^2^ | the smaller the better | 115.614 |  |
| df | the bigger the better | 59 |  |
| χ^2^/df | <3 | 1.960 | ideal |
| GFI | >0.9 | 0.960 | ideal |
| AGFI | >0.9 | 0.939 | ideal |
| RMSEA | <0.08 | 0.047 | ideal |
| CFI | >0.9 | 0.986 | ideal |
| TLI(NNFI） | >0.9 | 0.982 | ideal |

GFI, goodness of fit index; RMSEA, root mean square error of approximation; CFI, comparative fit index; TLI, Tucker–Lewis Index.
